# Benefit of Watching a Live Visual Inspection of the Cervix With Acetic Acid and Lugol Iodine on Women’s Anxiety: Randomized Controlled Trial of an Educational Intervention Conducted in a Low-Resource Setting

**DOI:** 10.2196/cancer.9798

**Published:** 2019-05-13

**Authors:** Roxane Camail, Bruno Kenfack, Phuong Lien Tran, Manuela Viviano, Pierre-Marie Tebeu, Liliane Temogne, Mohamed Akaaboune, Eveline Tincho, Joel Mbobda, Rosa Catarino, Pierre Vassilakos, Patrick Petignat

**Affiliations:** 1 University of Geneva Geneva University Hospitals Gynecology Division Geneva Switzerland; 2 University of Dschang Department of Biomedical Sciences Dschang Cameroon; 3 Yaoundé University Hospital Faculty of Medicine and Biomedical Sciences Yaoundé Cameroon; 4 Geneva Foundation for Medical Education and Research Geneva Switzerland

**Keywords:** cervical cancer, papillomavirus infections, acetic acid, Lugol’s iodine, anxiety

## Abstract

**Background:**

Women undergoing pelvic examination for cervical cancer screening can experience periprocedural anxiety.

**Objective:**

The aim of this study was to assess the anxiety level experienced by women undergoing a visual inspection with acetic acid and Lugol iodine (VIA and VILI) examination, with or without watching the procedure on a digital screen.

**Methods:**

This prospective randomized study took place in the district of Dschang, Cameroon. A previous cervical cancer screening campaign tested women aged between 30 and 49 years for human papillomavirus (HPV). HPV-positive women were invited for the 12-month follow-up control visit, including a VIA/VILI examination. During that visit, we recruited women to participate in this study. Before the examination, participants were randomized in a 1:1 ratio to a control group (CG) and an intervention group (IG). Women in both groups underwent a pelvic examination and were verbally informed about the steps undertaken during the gynecological examination. The IG could also watch it live on a tablet screen. Women’s anxiety was assessed before and immediately after the examination, using the Spielberger State-Trait Anxiety Inventory (STAI). A paired *t* test was used to compare the mean STAI score for each question before and after VIA/VILI while a nonpaired, 2-sided *t* test was used to compare the mean differences of the STAI score between the 2 study groups.

**Results:**

A total of 122 women were randomized in the study; 4 of them were excluded as they did not undergo the pelvic examination, did not answer to the second STAI questionnaire because of personal reasons, or the cervix could not be properly visualized. Thus, the final sample size consisted of 118 patients of whom 58 women were assigned to the CG and 60 to the IG. The mean age was 39.1 (SD 5.2) years. Before the examination, the mean (SD) STAI score was 33.6 (SD 10.9) in the CG and 36.4 (SD 11.8) in the IG (*P*=.17). The STAI score after pelvic examination was significantly reduced for both groups (CG: 29.3 [SD 11.2]; IG: 28.5 [SD 12.0]). Overall, the difference of the STAI scores before and after the pelvic examination was lower in the CG (4.2 [SD 9.0]) than in the IG (7.9 [SD 14.3]), although the difference was not significant (*P*=.10). However, the women’s emotional state, such as *I feel secure* and *I feel strained*, was improved in the IG as compared with the CG (CG: *P*=.01; IG: *P*=.007).

**Conclusions:**

Watching the VIA/VILI procedure in real time improved the women’s emotional state but did not reduce the periprocedural anxiety measured by the STAI score. Furthermore, larger studies should assess women’s satisfaction with watching their pelvic examination in real time to determine whether this tool could be included in VIA/VILI routine practice.

**Trial Registration:**

ClinicalTrials.gov NCT02945111; http://clinicaltrials.gov/ct2/show/NCT02945111

## Introduction

### Background 

Persistent human papillomavirus (HPV) infection is a major factor of cervical cancer (CC), which is the leading cause of cancer- related death in women in South Africa [1]. The lack of policies and resources for CC prevention in low- and medium-income countries (LMICs) is responsible for a high number of CC cases [2]. The updated 2012 World Health Organization (WHO) guidelines recommend the use of visual inspection with acetic acid (VIA) as a primary CC screening tool in LMICs, a strategy that entails a pelvic examination performed by an experienced physician. The WHO also recommends HPV-based primary screening with or without VIA triage for HPV-positive women [3].

Evidence supports that women undergoing pelvic examination can experience anxiety. This distressful feeling can be experienced before the examination (especially when it follows a pathological screening test result), during the examination, and up to several weeks after it [4,5]. The negative emotional responses experienced by patients that accompany the pelvic examination derive mainly from a poor understanding of the anatomy and a lack of knowledge about CC prevention procedures, which lead women to think that the purpose of screening is to detect cancer rather than to prevent it. Several studies observed that the high levels of stress associated with pelvic examinations could result in an exacerbation of procedure-related discomfort, which could discourage women from undergoing the procedure and induce low patient compliance [6,7].

As low compliance is a major barrier limiting the screening programs’ effectiveness, interventions were proposed to reduce the examination-related anxiety [8,9]. Among these, watching the examination in real time on a digital screen, giving women a better understanding of their anatomy, has shown to decrease women’s anxiety in some cases [10].

### Objectives

The aim of this study was to assess the anxiety level experienced by women undergoing a gynecological examination for VIA and visual inspection with Lugol iodine (VILI) while watching the procedure on a digital screen and to compare it with that of women who underwent the examination with no visual support.

## Methods

### Study Population and Setting

This prospective randomized study took place in September 2016 in the district of Dschang. Dschang is a city located in the West Province of Cameroon, with an estimated 200,000 inhabitants. A CC screening campaign was previously carried out in the Hospital of the District of Dschang in collaboration with the Geneva University Hospitals between July and October 2015, recruiting women aged between 30 and 49 years, living in Dschang and its surroundings. HPV-positive participants were invited for a 6- and 12-month follow-up visit to assess the disease status, and participants at the 12-month visit were invited to participate in this substudy. An inclusion protocol has already been previously reported [11]. The study was approved by the Central Ethics Committee on Human Research of the Geneva University Hospitals (approval number: CER 15-068) and the Ministry of Health of Cameroon [11], and the trial was registered on ClinicalTrials.gov with the identifier NCT02945111.

### Study Design and Intervention

Participants were thoroughly informed about the study and gave their written informed consent before participation. Enrolled participants were randomized in a 1:1 ratio into 2 groups: control group (CG) and intervention group (IG).

Enrolled participants, as a part of the follow-up visit, underwent a VIA and VILI examination, during which the physician took a cervical sample for cytology and HPV testing. Women in the CG underwent routine pelvic examination as described above. They were verbally informed about the steps undertaken during the examination. Women in the IG were given verbal information about the gynecological examination while they underwent the pelvic examination and also while watching it live on a tablet screen. With the help of the local study investigators, all women filled out a validated questionnaire to determine their anxiety level both before and after the pelvic examination. To avoid potential bias before the examination and the participants’ randomization, the tablet was placed on a table when it was not being used and picked up by the examiner only at the time of the pelvic examination for patients in the IG.

In the IG, the examiners took a picture of each step of the pelvic examination with a mobile phone camera (Samsung Galaxy S3, Samsung). This device, which was chosen for its high-quality camera (16 megapixels with autofocus and flash functions), allows highly precise and detailed visualization of the cervix after zooming and focusing in on the target. Photographs were obtained at a distance of 10 to 15 cm from the cervix, with 3.3 to 3.8x optical zoom in the flash mode. The smartphone was fixed on a tripod to improve the stability and quality of the images. The images were transmitted directly from the smartphone to the tablet, a Samsung Galaxy Tab (Samsung), using Bluetooth and a specifically designed app that enabled simultaneous communication between the 2 devices. Thus, women in this group could watch the pictures taken throughout the examination in real time. Image viewing was accompanied by the clinicians’ explanations on the anatomy (ectropion, dysplasia, nulliparous cervix, and multiparous cervix) and the procedure (with an interpretation of the VIA/VILI assessment).

### Spielberger State-Trait Anxiety Inventory Index

The anxiety was measured by asking participants in the two groups to complete the Spielberger State-Trait Anxiety Inventory (STAI) both before and immediately after the pelvic examination. The STAI is a standardized questionnaire created by Spielberger in 1983, broadly used and validated in psychology and in many medical fields [9]. It consists of 20 items describing various feelings and emotions that are present at that time. The following responses assess the intensity of current feelings *at this moment*: (1) not at all, (2) somewhat, (3) moderately so, and (4) very much so. Scoring should be reversed for anxiety-absent items. Once added up, the range of global scores is 20 to 80, the higher score indicating greater anxiety. This interview was usually self-completed, given the cultural differences and the heterogeneity of the educational backgrounds, although the STAI was filled out with the help of a local Cameroonian team consisting of 2 interviewers. The questionnaire was presented in French, which is one of the 2 national languages. When completing the STAI before the examination, neither the women nor the examiners knew in which group the patient was going to be randomly assigned. Randomization was done immediately before the pelvic examination, once the first STAI had been completed.

### Sample Size and Randomization

A Web-based statistical software [12] was used to generate the randomization list, with randomly permuted participants’ blocks of varying size (4, 6, and 8). This method made sure that the 122 participants were randomly attributed to either the CG or the IG while maintaining a balance across the 2 study groups. A further level of randomization consisted of using blocks of varying sizes. On the basis of this list, consecutively numbered, sealed opaque envelopes containing the group allocation were prepared. When a new participant gave her consent to participate in the study, and after having completed the first STAI questionnaire, the study investigator opened the next available envelope. We had assumed that 30% of patients in the CG would report an anxiety score ≥30, and we estimated to observe an 85% reduction of the overall anxiety levels in the IG (about 4.5% of patients with a score ≥30 in the IG). We estimated that 61 women were needed in each group to have an 85% power to detect a difference between groups with a 2-sided level of significance of .05 and while accounting for 30% of dropouts.

### Medical Chart

A secured, electronic medical chart using the secuTrial database (interActive Systems GmbH) including the sociodemographic and medical information (HPV test, VIA/VILI results, and cervical images) was created to register and retrieve the participants’ data.

### Statistical Analyses

Data were analyzed with the use of a statistical software package (Stata statistical software, release 14, StataCorp). Analyses were conducted according to the per-protocol principle. The paired *t* tests and Wilcoxon signed rank tests were used to compare the mean STAI score for each question before and after VIA/VILI. A nonpaired, 2-sided *t* test was used to compare the mean differences of the STAI score between the 2 study groups, as these results concern 2 independent populations of the study. The 2-sided chi-square test, the Fisher exact probability test, and the *t* test were used, where appropriate, to test the relationship between the patients’ sociodemographic and clinical characteristics and the STAI score both before and after the VIA/VILI examination.

## Results

### Study Design

**Figure 1:**
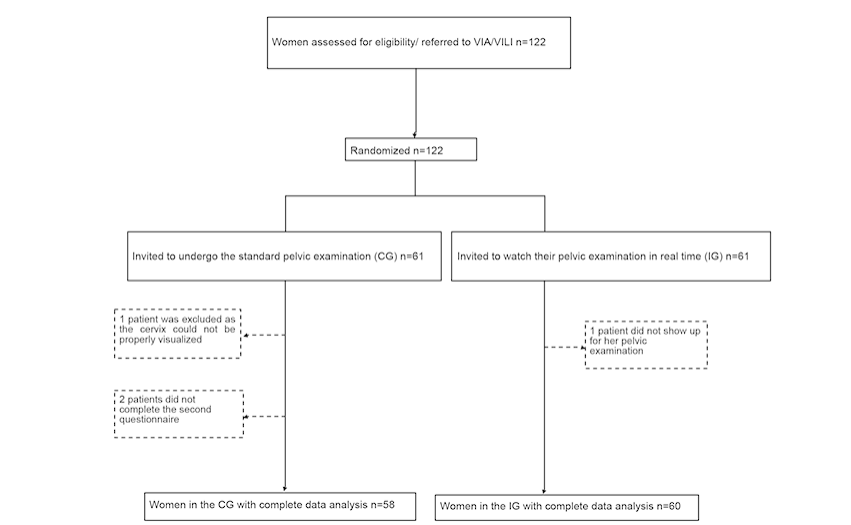
Flow chart. CG: control group; IG: intervention group; VIA: visual inspection with acetic acid; VILI: visual inspection with Lugol iodine.

This study took place in September 2016. A total of 122 women were included in the study; of these, 4 were excluded after having been randomized. The reasons for exclusion were as follows: 3 women did not undergo the pelvic examination or did not answer to the second STAI questionnaire because of personal reasons and 1 woman was excluded because the cervix could not be properly visualized during the pelvic examination. The final sample size thus consisted of 118 patients, of which 58 women were assigned to the CG and 60 to the IG (the study design is reported in Figure 1).

### Study Population and Setting

The mean (SD) age of the participants was 39.1 (SD 5.2) years. A total of 38 out of 58 (65%) women and 40 out of 60 (67%) women had part- or full-time employment in the CG and in the IG, respectively. As women were randomized, the 2 groups did not differ with regard to sociodemographic and clinical characteristics. Participants’ characteristics are summarized in Table 1. The primary CC screening campaign took place between July and August 2015. Only women who were HPV positive at the primary screening campaign were called back in September 2016 and were, therefore, invited to take part in this study. The HPV status as reported in Table 1 refers to the HPV status obtained at the follow-up visit that took place in September 2016.

### Spielberger State-Trait Anxiety Inventory Index

**Figure 2:**
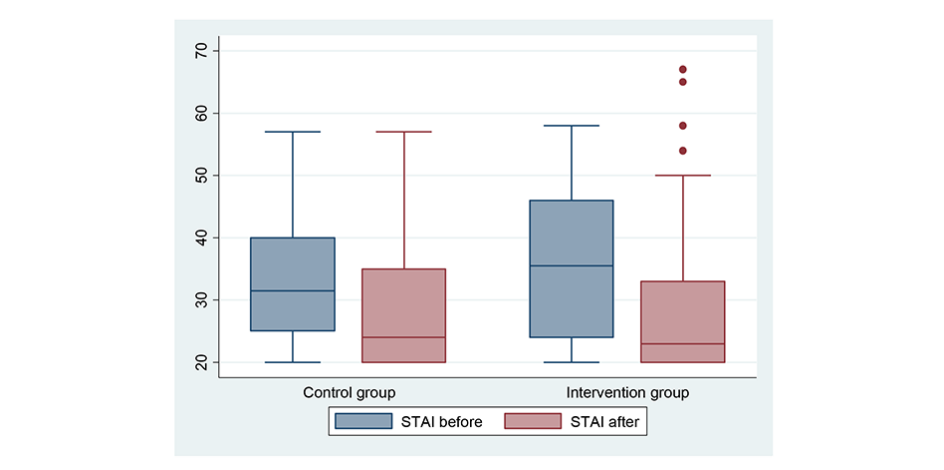
Box plot comparing anxiety between the control group and the intervention group. STAI: Spielberger State-Trait Anxiety Inventory.

Before the examination, the mean (SD) STAI score was 33.6 (SD 10.9) in the CG and 36.4 (SD 11.8) in the IG (*P*=.17). The mean STAI scores for each question before and after the pelvic examination are reported in Table 2. The total STAI score after the examination significantly decreased in both groups; in the CG, the mean score after examination was 29.3 (SD 11.2; *P*=.001) and in the IG, the STAI score dropped from 36.4 (SD 11.8) to 28.5 (SD 12.0; *P*<.001). The mean STAI scores before and after the pelvic examination in the CG and IG are illustrated in Figure 2. There was no particular reason to justify the presence of the 4 outlier cases in the IG having a higher STAI score than the rest of the participants in the same group after having undergone the pelvic examination. These 4 women were aged 38.9 (SD 5) years, they all had a full-time employment, and they had a mean of 4.1 (SD 1.1) children. These participants had a similar STAI score before and after the examination and did not increase their fear after the pelvic examination.

Overall, the difference of the STAI scores before and after the pelvic examination was higher in the IG (7.9 [SD 14.3]) than in the CG (4.2 [SD 9.0]), although the difference was not significant (*P*=.10). Questions such as *I feel secure* (number 2) and *I feel strained* (number 4) obtained a significantly higher score reduction among women in the IG when compared with those in the CG (0.1 [SD 1.1] in the CG and 0.7 [SD 1.2] in the IG, *P*=.007, for question 2 and 0.2 [SD 0.9] in the CG and 0.7 [SD 1.1] in the IG, *P*=.01, for question 4). Table 3 reports the comparison between the difference in STAI scores before and after the pelvic examination.

We found that women in both groups were less anxious if they had not been treated with thermocoagulation (*P*<.001) during the pelvic examination and if the VIA/VILI assessment had turned out to be nonpathological (*P*=.04). Results showed no other significant association.

**Table 1:** Sociodemographic and clinical characteristics of the study population

Variable			Control group (n=58)		Intervention group (n=60)	
**Sociodemographic characteristics, mean (SD)**						
	Age (years)			39.7 (5.2)		38.4 (5.2)
	Parity^a^			3.8 (1.9)		4.3 (1.8)
	**Marital Status, n (%)**					
		Single	3 (5)		4 (7)	
		With a partner	55 (95)		56 (93)	
	**Education level, n (%)**					
		None	11 (19)		1 (2)	
		Elementary school	1 (2)		11 (18)	
		Apprenticeship	33 (56)		3 (5)	
		High school	13 (22)		38 (63)	
		University	—^b^		7 (12)	
	**Employment status, n (%)**					
		Employed	38 (65)		40 (67)	
		Farmer	2 (3)		4 (7)	
		Housewife	15 (26)		13 (22)	
		Other	3 (5)		3 (5)	
**Clinical characteristics**						
	**HPV^c^** **test result^d^, n (%)**					
		Negative	41 (71)		36 (60)	
		HPV-16	—		2 (3)	
		HPV-18/45	4 (7)		3 (5)	
		Other hrHPV^e^	12 (21)		19 (32)	

^a^ Parity: number of pregnancies ended at a viable gestational age.

^b^Absence of corresponding data.

^c^HPV: human papillomavirus.

^d^There was one missing value in the control group’s human papillomavirus test results.

^e^hrHPV: high-risk human papillomavirus.

**Table 2:** Mean Spielberger State-Trait Anxiety Inventory scores in each study group.

Study question	Control group (n=58), mean (SD)			Intervention group (n=60), mean (SD)		
	Before pelvic examination	After pelvic examination	*P* value	Before pelvic examination	After pelvic examination	*P* value
1. I feel calm	1.7 (0.9)	1.7 (1.0)	.90	2.1 (1.0)	1.3 (0.8)	<.001
2. I feel secure	1.7 (0.9)	1.6 (1.0)	.62	2.1 (1.0)	1.5 (1.0)	<.001
3. I feel tense	1.5 (0.8)	1.3 (0.7)	.29	1.9 (1.0)	1.4 (0.9)	.002
4. I feel strained	1.4 (0.8)	1.2 (0.6)	.16	1.9 (1.0)	1.2 (0.7)	<.001
5. I feel at ease	1.7 (1.0)	1.4 (0.8)	.008	1.9 (1.0)	1.5 (1.0)	.03
6. I feel upset	1.7 (1.0)	1.3 (0.7)	.005	1.9 (1.0)	1.5 (1.0)	.02
7. I am presently worrying over possible misfortunes	1.6 (0.9)	1.3 (0.8)	.03	1.7 (1.0)	1.5 (1.1)	.23
8. I feel satisfied	1.8 (1.0)	1.6 (1.0)	.12	1.7 (1.0)	1.5 (1.0)	.15
9. I feel frightened	1.5 (0.9)	1.5 (0.9)	.74	1.7 (1.1)	1.6 (1.0)	.25
10. I feel uncomfortable	2.1 (1.3)	1.5 (1.0)	.002	1.9 (1.1)	1.4 (1.0)	.01
11. I feel self-confident	1.6 (0.9)	1.3 (0.8)	.08	1.7 (0.9)	1.4 (0.9)	.07
12. I feel nervous	1.4 (0.8)	1.2 (0.6)	.06	1.6 (0.9)	1.4 (1.0)	.27
13. I feel jittery	1.6 (0.9)	1.4 (0.9)	.09	1.7 (1.1)	1.4 (0.9)	.02
14. I feel indecisive	1.6 (0.9)	1.4 (0.8)	.12	1.7 (1.0)	1.3 (0.8)	.004
15. I am relaxed	1.8 (1.1)	1.6 (1.0)	.27	2.0 (1.0)	1.6 (1.1)	.03
16. I feel content	2.0 (1.1)	1.6 (1.0)	.002	1.7 (1.1)	1.4 (1.0)	.04
17. I am worried	1.9 (1.0)	1.5 (0.8)	.004	1.8 (1.0)	1.5 (1.0)	.10
18. I feel confused	1.6 (0.9)	1.3 (0.6)	.03	1.6 (0.9)	1.1 (0.5)	<.001
19. I feel steady	2.2 (1.0)	2.1 (1.1)	.46	2.1 (1.0)	1.6 (1.0)	.001
20. I feel pleasant	1.4 (0.8)	1.4 (0.9)	.47	1.8 (1.0)	1.6 (1.0)	.37
Total Spielberger State-Trait Anxiety Inventory score	33.6 (10.9)	29.3 (11.2)	.001	36.4 (11.8)	28.5 (12.0)	<.001

**Table 3:** Difference in Spielberger State-Trait Anxiety Inventory scores before and after the pelvic examination in each study group.

Study question	Control group (n=58), mean (SD)	Intervention group (n=60), mean (SD)	*P* value
1. I feel calm	−0.02 (1.1)	−0.7 (1.2)	.002
2. I feel secure	−0.1 (1.1)	−0.7 (1.2)	.007
3. I feel tense	−0.1 (1.0)	−0.5 (1.2)	.06
4. I feel strained	−0.2 (0.9)	−0.7 (1.1)	.01
5. I feel at ease	−0.3 (0.9)	−0.4 (1.2)	.91
6. I feel upset	−0.3 (0.9)	−0.5 (1.5)	.58
7. I am presently worrying over possible misfortunes	−0.3 (1.0)	−0.2 (1.2)	.58
8. I feel satisfied	−0.2 (0.9)	−0.2 (1.2)	.83
9. I feel frightened	−0.03 (0.8)	−0.2 (1.1)	.46
10. I feel uncomfortable	−0.6 (1.3)	−0.5 (1.4)	.68
11. I feel self-confident	−0.2 (0.9)	−0.3 (1.1)	.75
12. I feel nervous	−0.2 (0.8)	−0.2 (1.2)	.83
13. I feel jittery	−0.2 (0.8)	−0.4 (1.1)	.38
14. I feel indecisive	−0.2 (0.8)	−0.5 (1.2)	.12
15. I am relaxed	−0.2 (1.3)	−0.4 (1.4)	.39
16. I feel content	−0.4 (1.0)	−0.4 (1.4)	.77
17. I am worried	−0.4 (1.1)	−0.3 (1.4)	.57
18. I feel confused	−0.3 (0.9)	−0.5 (1.0)	.19
19. I feel steady	−0.1 (1.2)	−0.6 (1.2)	.06
20. I feel pleasant	−0.1 (0.5)	−0.2 (1.4)	.27
Total Spielberger State-Trait Anxiety Inventory score	−4.2 (9.0)	−7.9 (14.3)	.10

## Discussion

### Principal Findings

This study conducted in Cameroon aimed to assess the effect of watching a live VIA/VILI examination on women’s anxiety. The direct visualization of the pelvic examination was not associated with a reduction of anxiety as measured by the STAI score. When asked to report their emotional state through questions such as *I feel strained* and *I feel secure*, women who watched their examination on a digital screen were less anxious than women who underwent standard pelvic examination while receiving only a verbal explanation. Women in the 2 groups were similarly anxious before the pelvic examination, perhaps because of the limited knowledge of the visual support’s use and its way of functioning. The overall anxiety score decreased after having undergone the gynecological exam for women in both groups, with no significant difference for women who underwent the examination with a visual support. This finding can be explained by the fact that women are generally nervous about the pelvic examination before it starts and that once the procedure has come to an end, their anxiety generally decreases, regardless of the presence of the visual support.

The findings in this study appear to be in contradiction with previous data obtained by Walsh et al [10], who reported a significant anxiety reduction in the group that watched their pelvic examinations in real time when compared with those who did not watch the examination. However, it is difficult to compare our results with those obtained by Walsh et al as the study design was different: although they assessed the impact of watching live colposcopy on anxiety at a follow-up visit, we quantified anxiety with the STAI immediately after the procedure. On the contrary, our results are similar to those obtained by Hilal et al [13] *,* who found no significant differences in anxiety ratings between the group of participants who viewed the procedure on a digital screen monitor and the CG.

Previous studies have found that pelvic examinations can significantly increase women’s anxiety, thus discouraging them from attending screening and follow-up visits. As the anxiety of women participating in CC screening may be high, the negative emotional response associated with the pelvic examination can affect self-esteem, thus resulting in mood disorders such as depression and irritability [5]. An understanding of their anatomy and the natural history of CC is, therefore, an essential step in increasing women’s trust in CC screening and follow-up.

### Strengths and Limitations

The strengths of this study are the randomized and prospective design and the use of a measurement method that has previously been validated in the literature. Although the STAI is a standardized and validated questionnaire for Western countries, limited evidence has evaluated its use in settings such as in sub-Saharan Africa. The use of an alternative tool to measure participants’ anxiety may therefore have yielded different results.

One limitation of this study is that it took place at a 12-month follow-up visit, which means that all women had already undergone a gynecological examination with a VIA/VILI assessment. It is therefore possible that, as all women had already undergone the procedure, any intervention to reduce anxiety would be less influential. Another limitation is the cultural difference that may influence the perception of anxiety, which makes it difficult to generalize our study results to the rest of the worldwide population, in particular to that of industrialized countries. A limitation consists in the fact that there was no multiple comparison adjustment for statistically significant findings. Finally, the interviewer knew in which group the women had been randomized after the examination. This aspect may have introduced a potential bias, as knowing the participant’s group allocation may have influenced the way in which the STAI questions were asked.

Similarly, the participants were aware of their group allocation during the pelvic examination, as well as when filling the STAI form after it. Such an unmasked allocation may have influenced the study’s final results.

### Conclusions

In conclusion, watching the VIA/VILI procedure in real time improved the women’s emotional state but did not reduce the periprocedural anxiety measured by the STAI score. Furthermore, larger studies should assess women’s satisfaction with watching their pelvic examination in real time to determine if this tool could be included in VIA/VILI routine practice. Moreover, further research should be focused on the effect on women’s anxiety when showing their cervical images immediately after the procedure rather than during it.

### Acknowlegments

This study was supported by the Geneva University Hospitals, Geneva, Switzerland, and the Hospital of the District of Dschang, Cameroon. The funders had no role in the study conception and design or in the manuscript preparation and submission.

## Appendix

Multimedia Appendix 1 CONSORT‐EHEALTH checklist (V 1.6.1).

## Abbreviations

CC: cervical cancer
CG: control group
HPV: human papillomavirus
IG: intervention group
LMIC: low- and medium-income country
STAI: Spielberger State-Trait Anxiety Inventory
VIA: visual inspection with acetic acid
VILI: visual inspection with Lugol iodine
WHO: World Health Organization
